# Polarization-Robust and Electrically Tunable Quasi-Bound States in the Continuum in C_4_-Symmetric Terahertz Metasurfaces for Refractive-Index Sensing

**DOI:** 10.3390/nano16211377

**Published:** 2026-10-28

**Authors:** Mingyu Geng, Qinghua Wang, Yunjing Ji, Jiancheng Lai, Zhenhua Li

**Affiliations:** School of Physics, Nanjing University of Science and Technology, Nanjing 210094, China; g202021154006@163.com (M.G.); jyunjing@njust.edu.cn (Y.J.); laijiancheng@njust.edu.cn (J.L.); lizhenhua@njust.edu.cn (Z.L.)

**Keywords:** Terahertz metasurface, quasi-bound state in the continuum, C_4_ symmetry, graphene tuning, multipolar decomposition, refractive-index sensing

## Abstract

Polarization sensitivity and limited tunability remain important challenges for high-Q terahertz (THz) metasurfaces based on quasi-bound states in the continuum (q-BICs), particularly when geometric perturbations are introduced to enable radiative coupling. Here, we numerically demonstrate a graphene-integrated all-dielectric THz metasurface that combines high-Q q-BIC resonances, polarization robustness, electrical tunability, and refractive-index sensing through a C_4_-symmetric supercell design. Each metamolecule consists of two orthogonal amorphous-silicon strips, in which a width perturbation δ introduces local geometric asymmetry and enables the symmetry-protected states to couple to free space, while the four metamolecules related by successive 90° rotations preserve the global C_4_ rotational symmetry. Two q-BIC modes are supported, with their Q factors exhibiting the characteristic inverse-square dependence on δ. Multipolar analysis reveals that the lower-frequency q-BIC is dominated by magnetic-quadrupole radiation, whereas the higher-frequency mode is primarily governed by toroidal-dipole radiation and exhibits weaker overall radiative leakage. Owing to the C_4_-symmetric arrangement, the resonance spectra remain nearly invariant for normally incident linearly polarized THz waves over polarization angles from 0° to 90°. Integration of monolayer graphene further enables continuous electrical modulation of the resonance contrast and Q factor by varying the Fermi level from 0 to 0.7 eV, while inducing only minor resonance-frequency shifts. For bulk refractive-index sensing, the two q-BIC modes achieve sensitivities of 345 and 475 GHz/RIU, with maximum figures of merit exceeding 800 and approaching 10^4^, respectively. These results provide a versatile strategy for integrating polarization-robust high-Q resonances, electrical reconfigurability, and high-performance refractive-index sensing in THz metasurfaces.

## 1. Introduction

Terahertz (THz) electromagnetic waves occupy the spectral region between microwaves and infrared radiation and offer low photon energies, characteristic molecular fingerprints, and appreciable penetration through many nonconductive materials. These properties support applications in biomedical sensing, nondestructive testing, short-range high-speed communications, and security screening [1,2,3,4]. However, the weak THz response of many natural materials and the bulky form factor of conventional optical components limit efficient, integrated control of THz waves [5]. Metasurfaces—planar arrays of subwavelength artificial resonators—provide a compact platform for tailoring the amplitude, phase, polarization, and propagation of electromagnetic waves [6,7,8]. Nevertheless, conventional THz metasurface resonances often exhibit substantial radiative loss, modest quality factors (Q factors), and limited near-field enhancement, restricting their performance in narrowband filtering and high-sensitivity sensing [9].

Bound states in the continuum (BICs) are eigenstates embedded in the radiation continuum but decoupled from the available far-field channels. In the ideal lossless limit, a BIC can exhibit a divergent radiative Q factor and strong field confinement [10,11]. Photonic BICs commonly arise through symmetry protection or destructive interference between radiation channels and therefore cannot be directly excited from the far field under the corresponding symmetry conditions. A controlled perturbation that weakly breaks the protecting symmetry converts an ideal BIC into a quasi-BIC with a finite but potentially high Q factor, enabling far-field excitation while retaining pronounced field enhancement. This mechanism has consequently attracted substantial interest in THz metasurfaces [12,13].

Symmetry-protected BICs are among the most widely used mechanisms for realizing high-Q THz resonances. Periodic metasurfaces with C_4_, C_2_, or related in-plane symmetries can support symmetry-protected BICs at the Γ point. A small symmetry-breaking perturbation opens an otherwise forbidden radiation channel and produces an observable quasi-BIC; in the perturbative regime, the radiative Q factor typically scales inversely with the square of the asymmetry [14,15]. Wang et al. demonstrated polarization-insensitive THz quasi-BIC responses using a C_4_-symmetric supercell, highlighting the role of rotational symmetry in controlling polarization dependence [16]. Kovalev et al. investigated the robustness and tunability of BICs in toroidal metasurfaces and clarified how near-field coupling affects the resonance frequency and radiative loss [17]. All-silicon apertures, diamond-shaped metallic apertures, and dielectric resonators have also expanded the available design space for THz quasi-BIC metasurfaces [18,19,20].

Active control of quasi-BIC resonances is important for reconfigurable THz devices. Representative approaches include microelectromechanical-system (MEMS) deformation, liquid-crystal reorientation, phase-change materials, and electrical tuning with two-dimensional materials [21,22,23,24]. Graphene is particularly attractive because electrostatic gating continuously changes its Fermi level and, hence, its complex THz sheet conductivity. Its rapid carrier response, low electrical power requirement, and compatibility with planar structures have motivated extensive use in tunable THz devices [25]. Zhang et al. reported a graphene-based dual-band quasi-BIC THz absorber with continuously adjustable resonance strength [26], whereas Yang et al. used MEMS cantilevers to reconfigure the symmetry breaking and radiative loss. Liquid-crystal- and VO_2_-based schemes provide complementary routes to active control [22,23].

The narrow linewidths and enhanced near fields of quasi-BIC modes can strengthen light–matter interactions, making them useful for refractive-index sensing and biomolecular detection [27,28,29]. Maleki and Fathi employed second-order toroidal quasi-BIC modes in an all-dielectric metasurface for refractive-index sensing in biochemical environments [30]. Wang et al. combined a quasi-BIC THz metasurface with microfluidics to detect trace organic analytes [31]. Continued optimization of resonance Q factor, sensitivity, and figure of merit (FOM) is advancing quasi-BIC-based THz sensing [32].

Most symmetry-broken quasi-BIC metasurfaces, however, employ anisotropic resonators and therefore respond strongly only to a prescribed incident polarization. This dependence complicates their use with sources of unknown or varying linear polarization. In addition, fixed geometries provide little post-fabrication control. A quasi-BIC platform that combines polarization-independent operation with electrical tunability would therefore improve the adaptability of THz resonant devices.

Here, we propose a graphene-integrated THz quasi-BIC metasurface based on a C_4_-symmetric supercell. Each metamolecule (MM) comprises orthogonal amorphous-silicon strips, and widening one strip introduces a local symmetry-breaking perturbation that enables two quasi-BIC resonances. Four MMs, rotated successively by 90°, form a supercell with restored C_4_ symmetry at the lattice level. This hierarchy separates the local perturbation required for quasi-BIC excitation from the macroscopic rotational symmetry responsible for the nearly polarization-independent response. A monolayer graphene sheet provides electrical control of the modal loss and resonance contrast through its gate-tunable Fermi level. We analyze the geometric scaling of the resonances, their polarization response and multipolar radiation, graphene-mediated tuning, and refractive-index sensing performance. The present study is based on full-wave electromagnetic simulations intended to establish the operating mechanism and evaluate the device response. Experimental implementation will require accurate fabrication of the symmetry-broken a-Si resonators, uniform transfer and gating of monolayer graphene over the patterned surface, and polarization-resolved THz spectroscopy. Accordingly, the conclusions reported here should be interpreted as numerical predictions pending experimental validation.

## 2. Device Design and Simulation Setup

**Figure 1:**
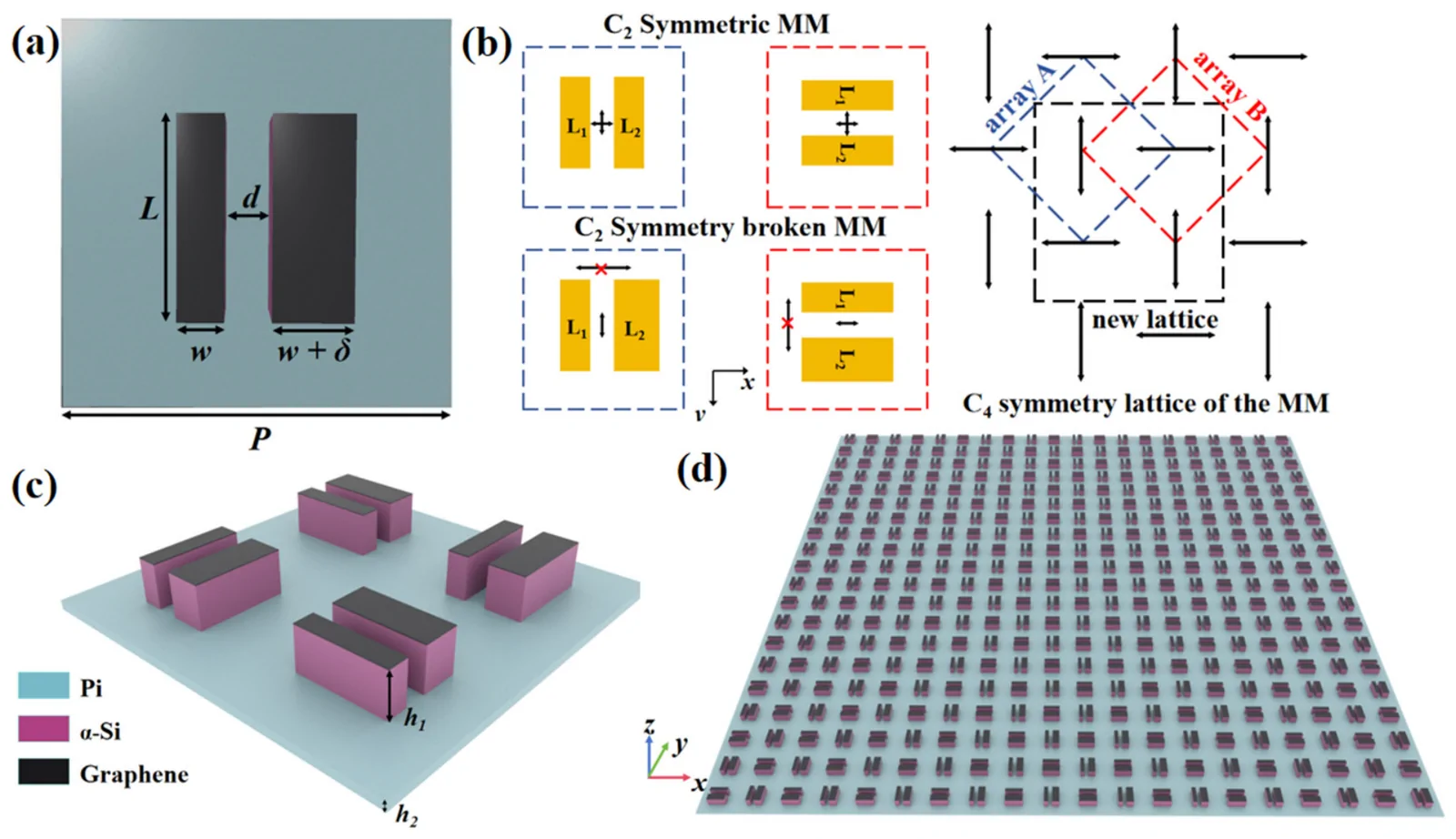
(**a**) Top view of a single MM. The geometric parameters are P = 100 μm, d = 26 μm, w = 11 μm, and L = 48 μm. (**b**) Individual MM and the C_4_-symmetric supercell formed by two interleaved arrays. Arrows indicate the MM orientations; red, blue, and black dashed lines outline arrays A and B and the resulting supercell, respectively. (**c**) Layered unit-cell geometry. (**d**) Schematic of the extended metasurface.

As shown in Figure 1a, each MM consists of two orthogonally arranged amorphous-silicon strips with rectangular cross sections. A local geometric asymmetry is introduced by increasing the width of the *x*-oriented strip by δ, where δ denotes the width perturbation. Although the two constituent strips are no longer geometrically equivalent for δ > 0, the fourfold rotational arrangement of the overall MM configuration is preserved. The MM orientation is defined by the axis of the widened strip, as illustrated in Figure 1b. Adjacent MMs are rotated by 90° and arranged as two interleaved subarrays: array A contains horizontally oriented MMs, whereas array B contains vertically oriented MMs. Four locally symmetry-broken MMs form a supercell that recovers C_4_ rotational symmetry at the supercell level. This distinction between local symmetry breaking and global C_4_ symmetry is central to the design. The consequences of the supercell symmetry can be expressed using the Jones transmission matrix. Consider a THz plane wave normally incident along the −z direction, with its electric field in the xy plane written as [16]

(1) $$E_{i}\left(r,t\right)=\left(\begin{array}{c} I_{x}\\ I_{y} \end{array}\right)e^{i\left(\mathrm{kz}-\mathrm{\omega t}\right)}$$

where ω is the angular frequency, k is the free-space wavenumber, and I_x_ and I_y_ are the complex incident-field amplitudes along x and y, respectively. The transmitted field is

(2) $$E_{t}\left(r,t\right)=\left(\begin{array}{c} t_{x}\\ t_{y} \end{array}\right)e^{i\left(\mathrm{kz}-\mathrm{\omega t}\right)}$$

The incident and transmitted fields are related by the 2 × 2 Jones transmission matrix:

(3) $$\left(\begin{array}{c} t_{x}\\ t_{y} \end{array}\right)=\left(\begin{array}{cc} T_{\mathrm{xx}} & T_{\mathrm{xy}}\\ T_{\mathrm{yx}} & T_{\mathrm{yy}} \end{array}\right)\left(\begin{array}{c} I_{x}\\ I_{y} \end{array}\right)$$

where T_ij_ denotes the complex transmission coefficient from j-polarized incidence to i-polarized transmission.

Under an in-plane rotation by an angle θ about the z axis, the coordinate-transformation matrix is

(4) $$D_{\theta }=\left(\begin{array}{cc} \mathrm{cos\theta} & \mathrm{sin\theta}\\ -\mathrm{sin\theta} & \mathrm{cos\theta} \end{array}\right)$$

The Jones matrix in the rotated basis is related to the original matrix by the similarity transformation:

(5) $$T_{\mathrm{new}}={D_{\theta }}^{-1}TD_{\theta }$$

For a C_4_-symmetric structure, a 90° rotation leaves the geometry invariant. Setting θ = π/2 therefore imposes the following constraint:

(6) $$D_{\pi /2}=\left(\begin{array}{cc} 0 & 1\\ -1 & 0 \end{array}\right),T_{\mathrm{new}}=\left(\begin{array}{cc} T_{\mathrm{xx}} & T_{\mathrm{xy}}\\ -T_{\mathrm{xy}} & T_{\mathrm{xx}} \end{array}\right)$$

This constraint gives T_xx_ = T_yy_ and T_xy_ = −T_yx_. C_4_ symmetry alone therefore makes the co-polarized responses along x and y identical.To achieve complete invariance of transmitted intensity under arbitrary linear polarization, the cross-polarized terms must additionally vanish or remain negligibly small, which is inherently guaranteed by the reciprocal mirror-symmetric configuration of the structure and has been validated through full-wave numerical simulations. At δ = 0, the relevant eigenmodes are symmetry-incompatible with normally incident plane waves, so their coupling to the open radiation channels vanishes. The modes are consequently absent from the transmission spectrum in the idealized lossless limit and form symmetry-protected BICs.

As shown in Figure 1c, the structure comprises a polyimide substrate, amorphous-silicon (a-Si) resonators, and a monolayer graphene tuning sheet. The 5-μm-thick polyimide substrate has a relative permittivity of 3.5 at 1 THz [33] and provides mechanical support and electrical isolation. The a-Si resonators have thickness h_1_ = 22.5 μm and are modeled with a relative permittivity of 11.9 and negligible dielectric loss in the frequency range considered [34]. The refractive-index contrast confines the modal field predominantly within and around the dielectric resonators. Graphene is placed on top of the a-Si structure; electrostatic control of its Fermi level changes its complex sheet conductivity and thereby modifies the dissipative loading and, to a lesser extent, the resonance frequency. At T = 293 K, the THz response of graphene is dominated by intraband carrier dynamics and is described using the intraband Kubo conductivity [25]:

(7) $$\begin{array}{c} \sigma \left(\omega \right)=\frac{ie^{2}E_{F}}{\pi \hbar^{2}\left(\omega +\frac{i}{\tau }\right)} \end{array}$$

where e is the elementary charge and the Fermi level is specified in electrinos; ℏ is the reduced Planck constant, ω is the angular frequency, and the carrier relaxation time is τ = 0.5 ps. Figure 1d shows the extended metasurface formed by periodically repeating the C_4_ supercells in air.

All full-wave electromagnetic simulations are performed based on the frequency-domain finite integration technique for periodic electromagnetic structures.For the periodic unit cell, Floquet periodic boundary conditions are imposed along the x and y directions to replicate the infinite two-dimensional array. Perfectly matched layer (PML) boundary conditions are applied along the z direction (wave propagation direction) at both sides of the computational domain to eliminate spurious reflections from domain truncation. A linearly polarized plane wave is normally incident from the top port along the −z direction, and the transmission amplitude is extracted from the S-parameter as T(ν) = |S_21_|. The input and output ports are placed 50 μm away from the metasurface along the z axis to ensure full separation of the incident and reflected fields.

A tetrahedral mesh scheme is adopted for the entire computational domain. To strike a balance between numerical accuracy and computational efficiency, an adaptive mesh refinement strategy is implemented: the mesh is automatically refined in regions with intense field localization and steep field gradients, including the graphene layer, the sidewalls of the a-Si dielectric strips, and the gaps between adjacent resonant structures. The minimum mesh element size is constrained to be much smaller than the characteristic geometric dimensions of the structure, and the global mesh density satisfies at least four mesh cells per shortest wavelength in the terahertz band, which ensures convergence of both the resonant frequency and transmission amplitude.

Monolayer graphene is modeled as an infinitesimally thin conductive surface (rather than a bulk three-dimensional material) assigned with the complex surface conductivity defined by Equation (7), which accurately captures its two-dimensional electromagnetic response. This surface-conductivity modeling approach avoids numerical artifacts introduced by discretizing an atomically thin layer as a bulk volume, and improves both computational accuracy and efficiency.

**Figure 2:**
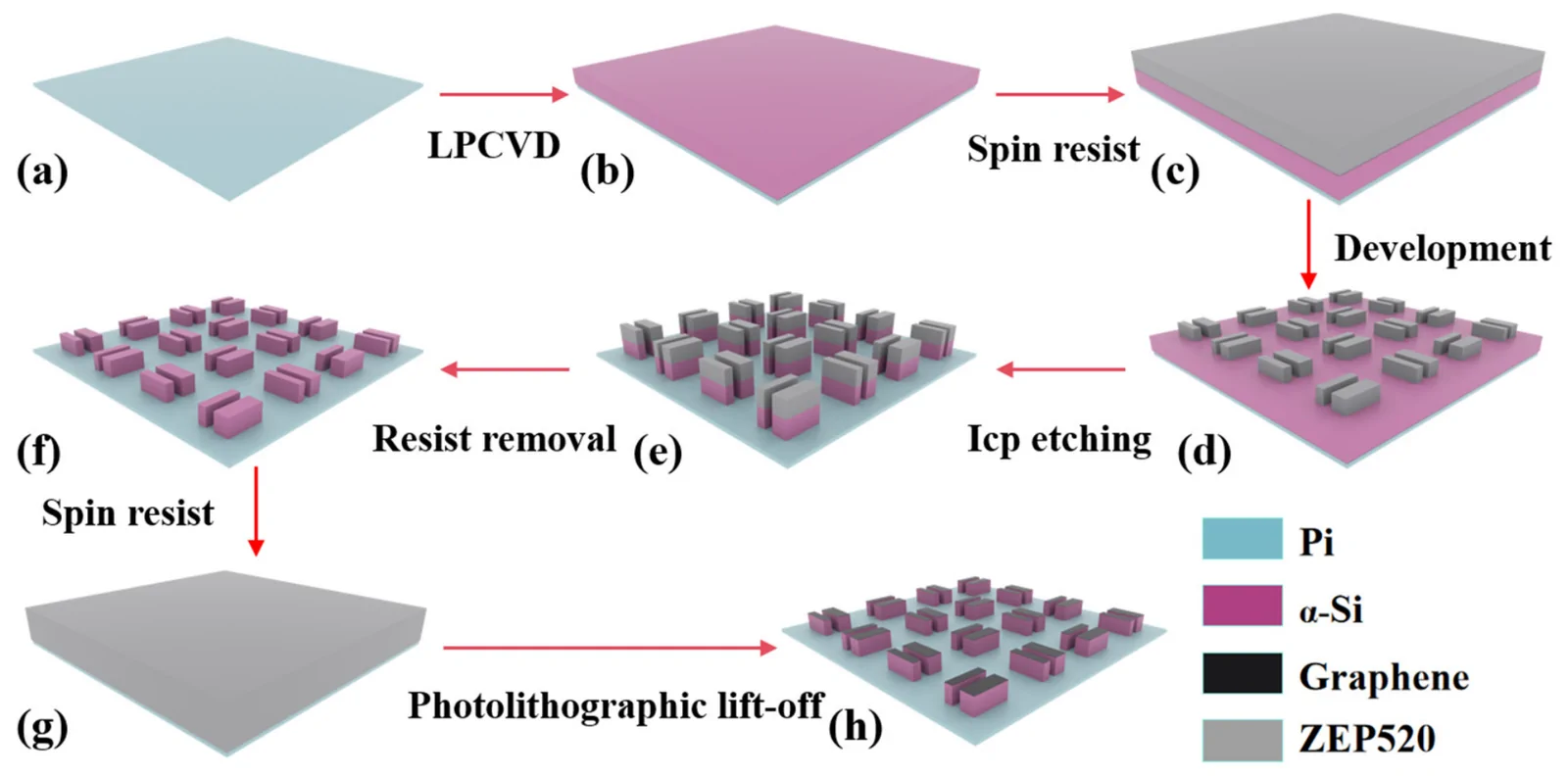
Process flow diagram for structural manufacturing. (**a**) Pretreated silicon carrier wafer with a spin-coated and cured polyimide (PI) thin film serving as the flexible substrate. (**b**) Low-temperature deposition of the hydrogenated amorphous silicon (a-Si:H) functional layer via plasma-enhanced chemical vapor deposition (PECVD). (**c**) Photolithography patterning to define the C_4_-symmetric resonator array and alignment marks on the photoresist layer. (**d**) Inductively coupled plasma (ICP) dry etching to transfer the patterned array into the a-Si:H layer, with the etching endpoint stopping at the PI substrate. (**e**) Fabricated all-dielectric metasurface base after photoresist removal and surface cleaning. (**f**) Conformal wet transfer of a monolayer CVD-grown graphene sheet onto the structured non-flat substrate with the support of a PMMA layer. (**g**) Patterned graphene structures precisely aligned to the underlying dielectric pillars, obtained by aligned photolithography and oxygen plasma etching. (**h**) Optional coplanar Ti/Au ohmic electrodes and bonding pads fabricated for electrical tuning of the graphene Fermi level.

The C_4_-symmetric all-dielectric graphene-integrated terahertz quasi-BIC metasurface proposed in this work can be fabricated via a standard micro–nano processing workflow, as illustrated in Figure 2.

A polyimide (PI) flexible substrate is selected as the device base. This material exhibits low absorption loss and high optical uniformity in the terahertz band, along with excellent mechanical flexibility, making it compatible with diverse application scenarios such as conformal surface sensing and wearable sensing systems.

Starting from the PI substrate, a layer of amorphous silicon (α-Si) thin film is first deposited on the substrate surface via low-pressure chemical vapor deposition (LPCVD) at low temperature. By tuning the deposition temperature, reaction pressure and gas flow rate, the film thickness and dielectric properties can be precisely controlled. Amorphous silicon features high refractive index and low loss in the terahertz frequency range, serving as an ideal dielectric material for constructing high-Q quasi-BIC resonant structures.

Subsequently, ZEP520 photoresist is uniformly spin-coated on the α-Si film. After pre-baking curing, the pre-designed periodic array pattern is transferred to the photoresist layer through photolithographic exposure and development, forming a patterned etching mask. On this basis, inductively coupled plasma (ICP) dry etching is performed to vertically etch the α-Si film using the photoresist as the mask, so as to accurately transfer the array structure to the amorphous silicon layer. By optimizing the etching gas composition, power and etching time parameters, dielectric pillar arrays with steep sidewalls and uniform morphology can be obtained, and the etching endpoint stops precisely at the PI substrate surface to avoid damage to the base. After etching, the residual photoresist is removed via oxygen plasma ashing combined with wet cleaning, yielding the all-dielectric metasurface base structure.

To achieve the patterned integration of graphene with precise alignment to the underlying dielectric pillar array, a photolithography-assisted lift-off process combined with conformal wet transfer is adopted, which is a well-established fabrication route for graphene-integrated terahertz metasurfaces [35]. First, a fresh layer of photoresist is spin-coated uniformly over the pre-fabricated all-dielectric metasurface with stepped topography. The target graphene regions are defined via photolithography, where an optical alignment system matches the photomask pattern to the underlying a-Si pillar array. The alignment accuracy can reach the sub-micrometer scale, which is sufficiently high for the 10-μm-scale geometric features in this work [36].

Afterwards, a monolayer graphene sheet grown by chemical vapor deposition (CVD) is transferred onto the patterned substrate via a polymer-supported wet transfer process. Benefiting from the flexibility of the polymer supporting layer, the graphene film can conformally attach to both the top surfaces and sidewalls of the dielectric pillars, avoiding tearing or suspended regions at the step edges that commonly occur with conventional rigid transfer methods [37].

Finally, the excess graphene and the overlying photoresist are removed by a standard lift-off process in organic solvent, leaving patterned graphene sheets precisely registered on top of the a-Si resonators. This completes the fabrication of the graphene-integrated flexible terahertz quasi-BIC metasurface.

The entire fabrication process adopts mature semiconductor micro–nano processing technologies with good process compatibility and repeatability, enabling large-area and high-precision device fabrication [38], which provides feasible technical support for subsequent experimental testing and sensing applications.

## 3. Results and Discussion

**Figure 3:**
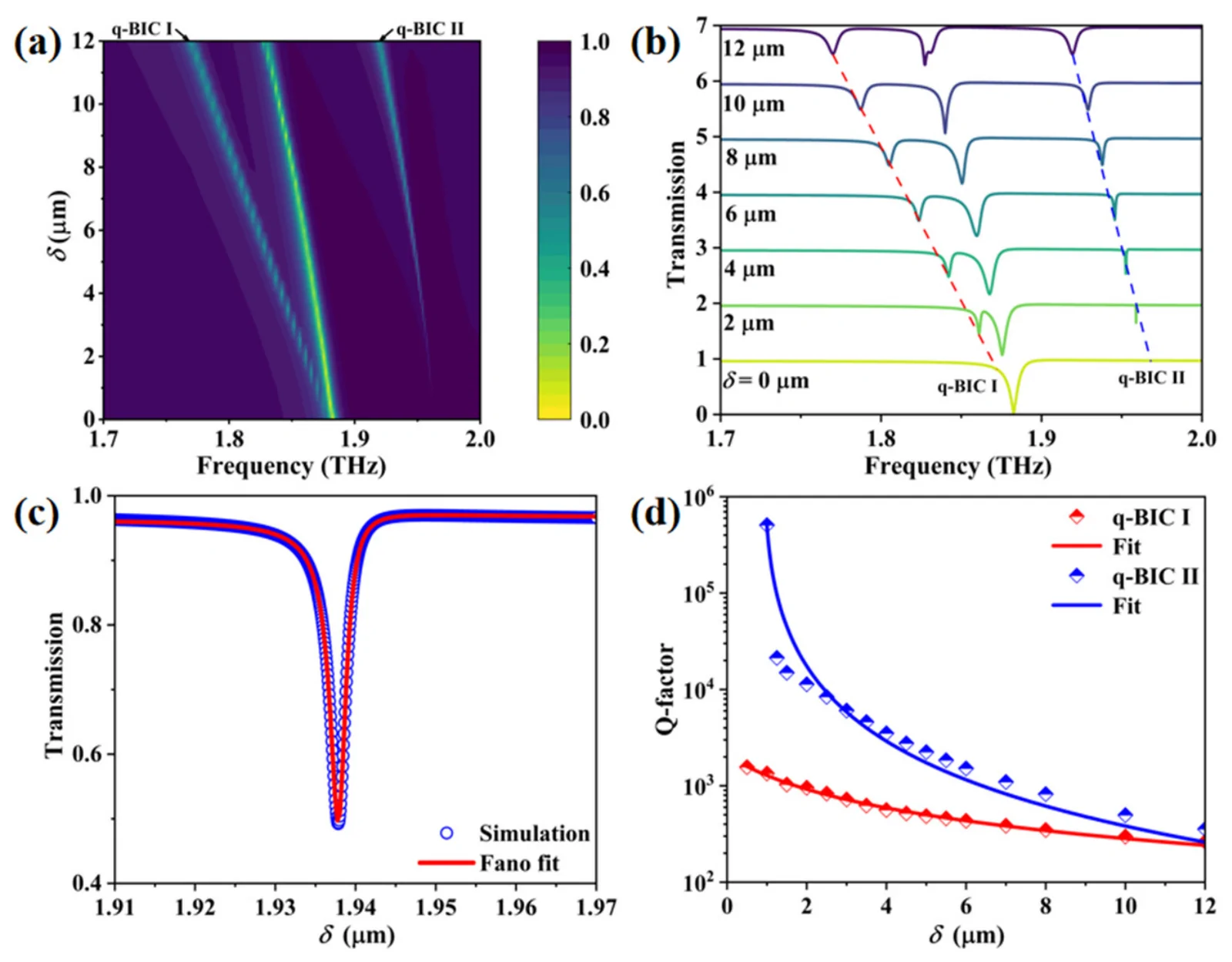
(**a**) Transmission map and (**b**) selected transmission spectra as δ increases.The red and blue dashed lines trace the frequency shifts of the transmission dips for q-BIC I and q-BIC II, respectively. (**c**) Simulated q-BIC II spectrum and Fano fit at δ = 8 μm. (**d**) Q factors of the two quasi-BIC modes as functions of the dimensional width perturbation δ.

Figure 3 shows the evolution of the simulated transmission spectrum with the dimensional width perturbation δ = 0–12 μm under x-polarized incidence. Figure 3a maps the transmission amplitude as a function of frequency and δ. At δ = 0 μm, each MM and the supercell retain C_4_ symmetry, and no pronounced resonance dip appears between 1.7 and 2.0 THz. In this symmetry-protected limit, the relevant eigenmodes are decoupled from normal-incidence radiation and therefore do not produce observable far-field features.

For δ > 0, the width perturbation introduces local geometric asymmetry into each MM, while the four-MM supercell preserves its global C_4_ rotational arrangement. As a result, two distinct transmission dips emerge, denoted q-BIC I at the lower frequency and q-BIC II at the higher frequency. An additional weaker and broader resonance feature is also visible between the two q-BIC modes at approximately 1.85 THz. This intermediate feature is a symmetry-allowed bright hybrid multipolar resonance that remains directly excitable even at δ = 0 μm; it does not originate from the perturbation-induced opening of a symmetry-protected dark state, and therefore does not follow the characteristic BIC-to-q-BIC evolution. Its broader linewidth and stronger far-field coupling also result in a substantially lower Q factor compared with the two q-BIC modes. Since this work focuses on high-Q resonances derived from symmetry-protected BICs, the quantitative analysis concentrates on q-BIC I and q-BIC II, while the field distribution and physical origin of the intermediate resonance are clarified for completeness.

With increasing δ, both q-BIC resonances become progressively more pronounced and broader, reflecting enhanced radiative coupling between the quasi-BIC modes and free space. Figure 3b presents the transmission spectra for δ = 0, 2, 4, 6, 8, 10, and 12 μm. At δ = 8 μm, both modes exhibit sufficient spectral contrast while retaining relatively narrow linewidths, providing a suitable compromise between far-field accessibility and resonant confinement. This value is therefore adopted for the subsequent analysis unless otherwise specified.

To quantitatively characterize the asymmetric spectral response, the simulated transmission spectrum is fitted using the Fano line-shape function [39]

(8) $$\begin{array}{c} T={\left|a_{1}+ia_{2}+\frac{b}{\omega -\omega_{0}+\mathrm{i\gamma}}\right|}^{2} \end{array}$$

where *a*_1_ and *a*_2_ denote the real and imaginary components of the resonant term, respectively; *b* is the resonant amplitude coefficient; *ω*_0_ is the resonance angular frequency; and *γ* represents the total damping rate. Figure 3c compares the simulated transmission spectrum of q-BIC II at δ = 8 μm with the corresponding Fano fit. The excellent agreement between the simulated and fitted spectra demonstrates that the asymmetric resonance can be accurately described by the Fano line-shape model.

Based on the fitted resonance parameters, the quality factor is evaluated as follows [40]:

(9) $$Q=\frac{\omega_{0}}{2\gamma }$$

Figure 3d summarizes the extracted Q factors of q-BIC I and q-BIC II as a function of δ. For both modes, Q decreases rapidly with increasing δ, consistent with the characteristic inverse-square scaling behavior of symmetry-protected quasi-BIC resonances in the perturbative regime. This behavior reflects the progressive opening of radiative leakage channels as the local geometric perturbation increases. Notably, q-BIC II maintains a substantially higher Q factor than q-BIC I throughout the investigated range. At δ = 2 μm, the Q factor of q-BIC II reaches the order of 10^5^, whereas that of q-BIC I is approximately 10^3^. Even at δ = 8 μm, q-BIC II retains a Q factor above 10^3^. The pronounced difference between the two modes indicates substantially weaker radiative leakage for q-BIC II, consistent with the multipolar radiation characteristics discussed below.

**Figure 4:**
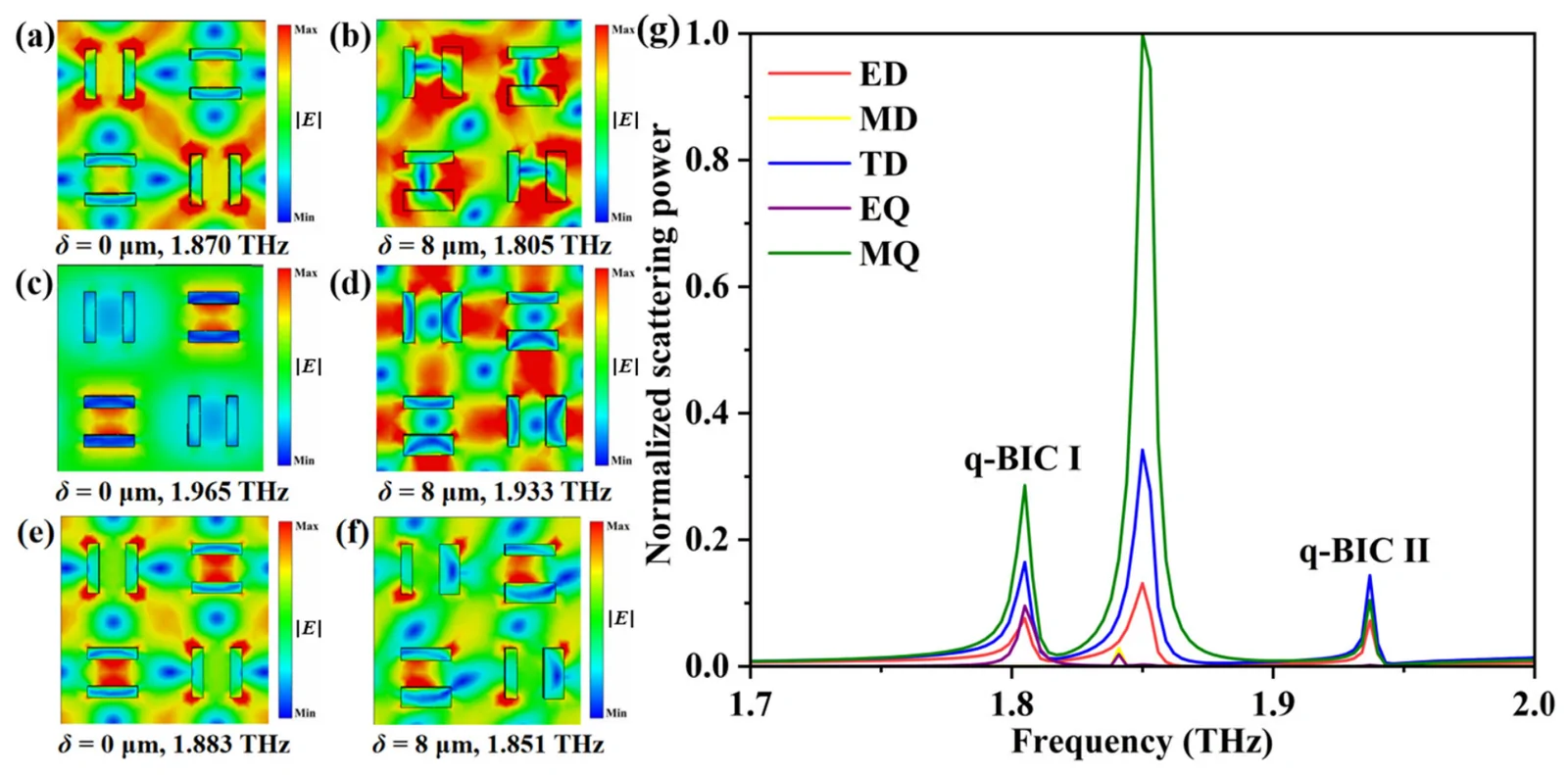
Electric-field distributions of (**a**) q-BIC I at δ = 0 μm and 1.870 THz, (**b**) q-BIC I at δ = 8 μm and 1.805 THz, (**c**) q-BIC II at δ = 0 μm and 1.965 THz, (**d**) q-BIC II at δ = 8 μm and 1.933 THz, (**e**) q-BIC II at δ = 0 μm and 1.883 THz, and (**f**) q-BIC II at δ = 8 μm and 1.851 THz. Panels (**a**–**f**) show the magnitude of the total electric field |*E*| at the z = h_1_ + h_2_ plane, with a linear color scale. (**g**) Normalized multipolar decomposition of the two quasi-BIC resonances at δ = 8 μm.

To elucidate the physical origin of the BIC-to-q-BIC transition, the evolution of the near-field distributions is analyzed together with the corresponding far-field multipolar radiation. For q-BIC I, the electric field at δ = 0 is strongly confined within the silicon resonators and the gap regions between their ends, as shown in Figure 4a, with only weak field extension into the surrounding medium. The symmetry-related field contributions of the four MMs suppress coupling to the radiation continuum under normal incidence, resulting in a symmetry-protected BIC state. When the local geometric perturbation is increased to δ = 8 μm, the field distribution becomes asymmetric and extends further into the surrounding region, as shown in Figure 4b. This redistribution relaxes the symmetry-imposed coupling restriction and opens a finite radiative channel, thereby transforming the ideal BIC into an observable q-BIC resonance.

A similar field evolution is observed for q-BIC II in Figure 4c,d. At δ = 0, the resonant field remains strongly confined, while the symmetry-related field contributions suppress coupling to the accessible free-space radiation channels. At δ = 8 μm, the local geometric perturbation redistributes the resonant field and enables finite coupling between the originally dark state and the incident THz wave. For the intermediate resonance at ~1.85 THz, Figure 4e,f show its field distributions at δ = 0 and δ = 8 μm, respectively. In contrast to the two q-BIC modes, this mode already exhibits substantial field leakage and far-field coupling at δ = 0, confirming its nature as a symmetry-allowed bright resonance rather than a symmetry-protected BIC. Its field pattern combines features of magnetic-quadrupole and toroidal-dipole topology, consistent with its hybrid multipolar character.

The multipolar decomposition in Figure 4g further reveals the radiation characteristics of the two q-BIC modes and provides a physical interpretation of their distinct dominant channels. For q-BIC I, the alternating electric-field polarity and end-localized hotspots of the four successively rotated meta-molecules correspond to anti-phase displacement-current loops and oppositely directed local magnetic moments. The global C_4_ arrangement strongly suppresses the net electric- and magnetic-dipole moments, whereas the spatially alternating magnetic moments retain a finite magnetic-quadrupole (MQ) moment. Once the local width perturbation breaks the symmetry of each meta-molecule, this MQ channel acquires finite radiative coupling and therefore dominates q-BIC I.

For q-BIC II, the phase arrangement of the fields in the four rotated meta-molecules supports a head-to-tail circulation of the local magnetic moments, equivalently a poloidal displacement-current pattern encircling the supercell. This topology produces a strong toroidal-dipole (TD) moment, while the C_4_ organization largely cancels the net ED and MD radiation. The remaining MQ contribution to q-BIC II arises from the finite local asymmetry and incomplete cancellation among the four meta-molecules. Because the TD-dominated mode couples more weakly to the far-field radiation continuum than the MQ-dominated q-BIC I mode, q-BIC II exhibits lower total radiated power, a narrower linewidth, and a substantially higher Q factor.

Taken together, the near-field and multipolar analyses show that the local geometric perturbation enables the BIC-to-q-BIC transition, whereas the distinct multipolar radiation characteristics—determined by the field topology within each MM and their rotational arrangement in the supercell—govern the different radiative losses and Q factors of the two modes.

**Figure 5:**
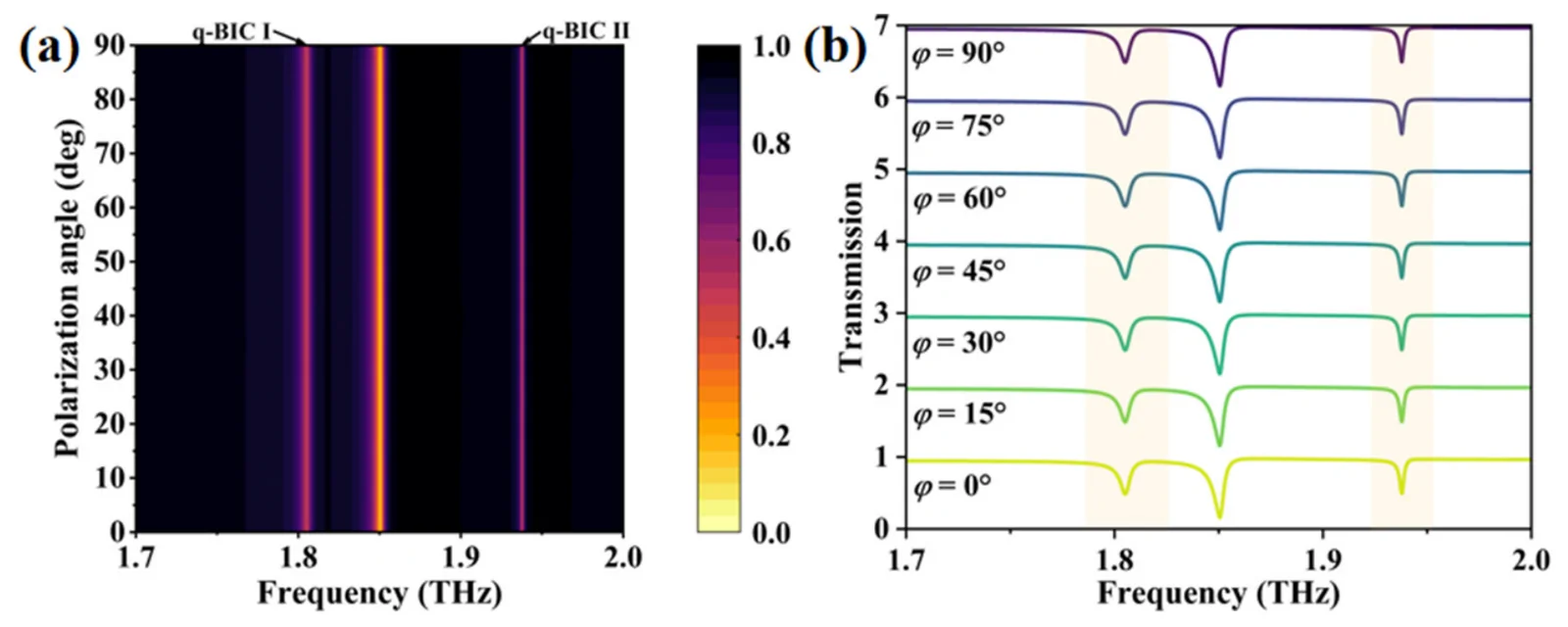
(**a**) Transmission map and (**b**) selected spectra for different incident linear-polarization angles.

The polarization dependence of the proposed metasurface was investigated by varying the incident linear polarization angle from 0° (x polarization) to 90° (y polarization) at δ = 8 μm. As shown in Figure 5a, both q-BIC I and q-BIC II remain nearly unchanged throughout the entire polarization range, with negligible variations in resonance frequency, transmission depth, and linewidth. The discrete spectra at polarization angles of 0–90° are further compared in Figure 5b, where the transmission curves nearly overlap over the investigated frequency range, confirming the polarization-insensitive response under normally incident linearly polarized THz waves. This polarization robustness originates from the C_4_-symmetric organization of the supercell. Although each locally perturbed MM exhibits an orientation-dependent response, the four MMs are related by successive 90° rotations, allowing their in-plane anisotropic responses to compensate at the supercell level. Consequently, the metasurface preserves nearly identical q-BIC characteristics for different linear polarization orientations. Such polarization insensitivity relaxes the requirement for precise alignment between the incident polarization and the resonator orientation, which is advantageous for practical THz sensing applications.

**Figure 6:**
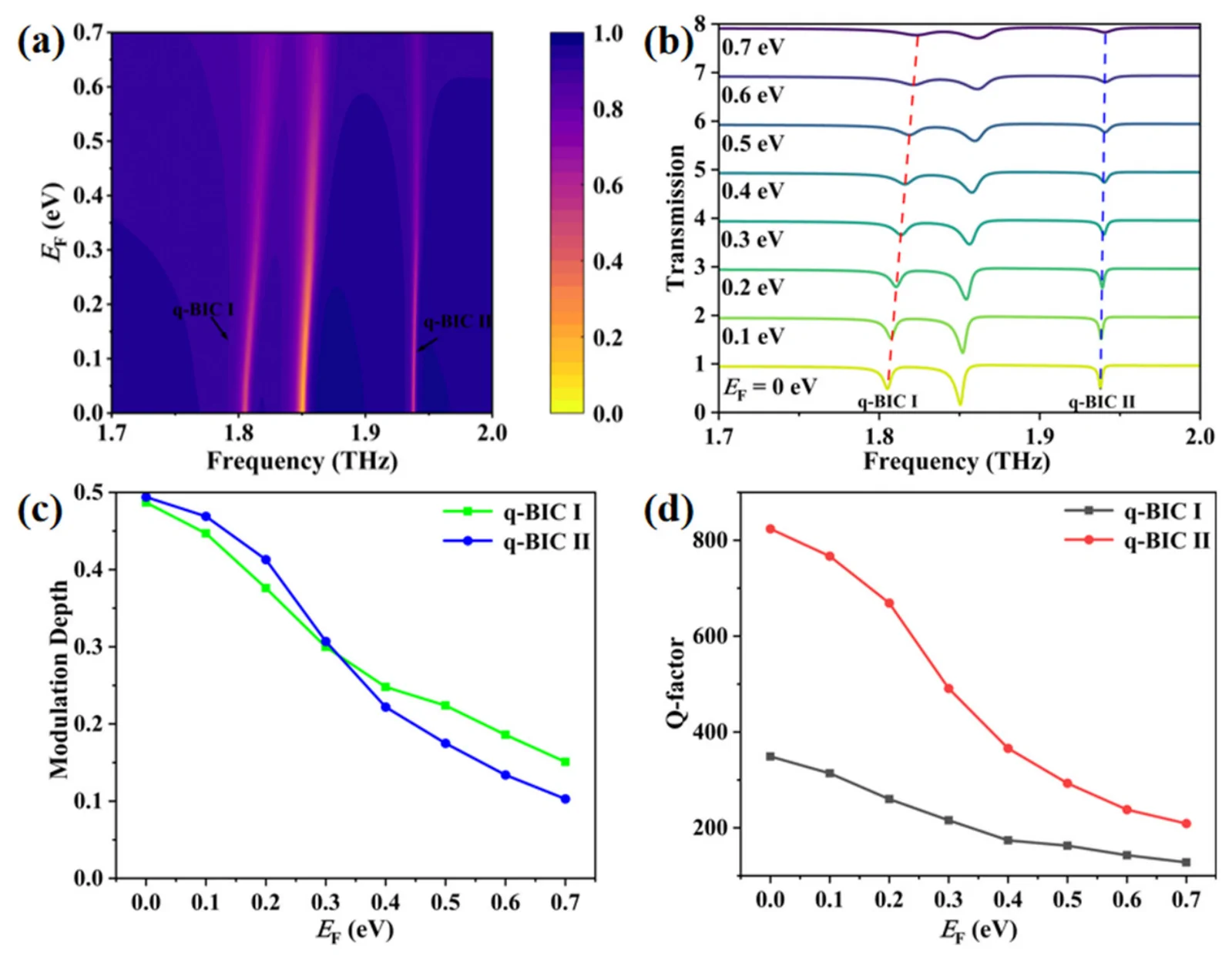
(**a**) Transmission map and (**b**) selected spectra for different graphene Fermi levels. (**c**) Modulation depth and (**d**) Q factor of the two quasi-BIC modes versus Fermi level.

To enable active electrical tuning, a monolayer graphene sheet is integrated with the silicon resonators. Variation in the graphene Fermi level modifies its complex THz sheet conductivity and consequently alters the electromagnetic loading of the q-BIC modes. At δ = 8 μm, increasing the Fermi level from 0 to 0.7 eV progressively reduces the depth of both transmission dips and slightly broadens their linewidths, while only a minor blueshift of the resonance frequencies is observed, as shown in Figure 6a. The graphene tuning therefore predominantly modulates the resonance amplitude and linewidth rather than producing a substantial frequency shift. The corresponding transmission spectra at Fermi levels from 0 to 0.7 eV in steps of 0.1 eV are compared in Figure 6b. At E_F_ = 0 eV, the graphene layer introduces relatively weak loading under the adopted conductivity model, resulting in the largest resonance contrast and narrowest linewidths. As EF increases, the enhanced graphene conductivity introduces additional dissipative loss, which weakens the field confinement and increases the total damping of the q-BIC modes. Consequently, the transmission dips become progressively shallower and broader. The comparatively small frequency shift can be understood from the interaction between the graphene sheet and the q-BIC fields. Because the resonant fields are predominantly localized within the a-Si resonators, their spatial overlap with the atomically thin graphene layer is limited. Within the intraband conductivity model, the real part of the graphene conductivity, Re(σ), is associated with dissipative loss, whereas its imaginary part, Im(σ), contributes to reactive electromagnetic loading. In the present structure, the limited field overlap suppresses the reactive perturbation of the resonant modes, whereas the additional dissipative loss remains sufficient to modify their resonance contrast and linewidth. This accounts for the predominantly amplitude-based modulation accompanied by only a weak shift in resonance frequency.

To quantify the electrically controlled variation in resonance contrast, the modulation depth (MD) is defined in terms of the off-resonant background transmittance *T_bg_* and the minimum transmittance *T_res_* at the resonance as follows [41]:

(10) $$\mathrm{MD}=\frac{T_{\mathrm{bg}}-T_{\mathrm{res}}}{T_{\mathrm{bg}}}$$

As shown in Figure 6c, the MD values of both q-BIC modes decrease monotonically with increasing Fermi level. The enhanced intraband conductivity of graphene introduces additional dissipative loading to the resonant system, thereby reducing the resonance contrast and producing progressively shallower transmission dips. This behavior confirms that electrostatic gating provides continuous control over the resonance amplitude through modulation of the graphene-associated loss. A similar trend is observed for the Q factors in Figure 6d, which gradually decrease as the Fermi level increases. Because the resonator geometry and the magnitude of the local geometric perturbation remain unchanged during Fermi-level tuning, the intrinsic radiative coupling channel is expected to remain approximately unchanged. The reduction in Q is therefore primarily attributed to the increased absorptive loss introduced by graphene. In contrast, varying δ directly modifies the radiative coupling strength and consequently produces a much stronger variation in Q. The two tuning parameters thus play distinct roles: δ predominantly governs the radiative leakage of the q-BIC modes, whereas the graphene Fermi level provides electrical control through additional material loss.

Overall, electrostatic tuning of graphene enables continuous modulation of the resonance contrast and Q factor while inducing only minor changes in the resonance frequencies. This provides an additional degree of freedom for electrically adjusting the operating contrast and bandwidth of the metasurface without modifying its physical geometry.

**Figure 7:**
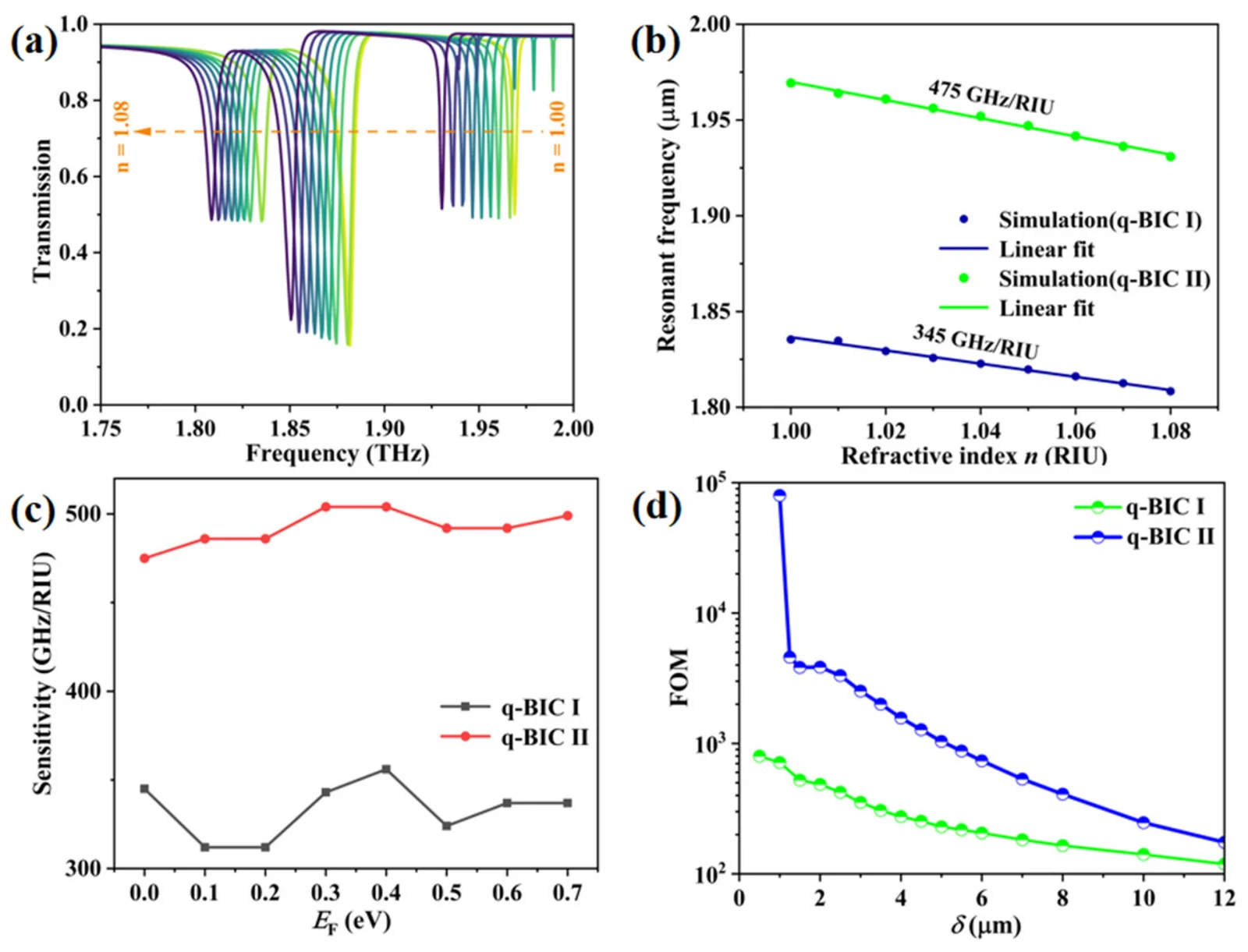
(**a**) Transmission spectra for surrounding refractive indices from 1.00 to 1.08. (**b**) Resonance frequency versus surrounding refractive index. (**c**) Sensitivity versus graphene Fermi level. (**d**) FOM versus the dimensional width perturbation δ. Unless otherwise indicated, δ = 8 μm.

The strong field confinement and narrow linewidths of q-BIC resonances provide favorable conditions for refractive-index sensing, as variations in the surrounding dielectric environment perturb the resonant modes and produce measurable spectral shifts [27]. The bulk refractive-index response of the proposed metasurface is therefore evaluated, together with the influence of graphene tuning on the sensing performance. Unless otherwise specified, δ = 8 μm. As shown in Figure 7a, both q-BIC resonances exhibit a clear redshift as the surrounding refractive index n increases from 1.00 to 1.08, while their resonance depths and asymmetric line shapes remain comparatively stable. The redshift originates from the increase in the effective refractive index experienced by the resonant modes. For a fixed resonator geometry, the resonance frequency approximately follows *f_res_*∝1/*n_eff_*; therefore, an increase in the surrounding refractive index increases the effective optical path length and shifts the resonances toward lower frequencies.

The bulk refractive-index sensitivity S is defined as the resonance-frequency shift per refractive-index unit [42]: *S* = |∆*f*/∆*n*|, where ∆*f* is the change in resonance frequency induced by a refractive-index variation ∆*n*, and the sensitivity is expressed in GHz/RIU. Figure 7b plots the resonance frequencies of q-BIC I and q-BIC II as functions of the surrounding refractive index. Linear fitting yields sensitivities of 345 GHz/RIU for q-BIC I and 475 GHz/RIU for q-BIC II over the investigated range of n = 1.00–1.08. The higher sensitivity of q-BIC II indicates a stronger interaction between its resonant field and the surrounding dielectric environment, consistent with a larger effective perturbative overlap of the mode with the sensing region.

Figure 7c shows the extracted sensitivities of the two modes as the graphene Fermi level varies from 0 to 0.7 eV. Only minor variations are observed, indicating that graphene tuning predominantly modifies the resonance contrast and linewidth while exerting a limited influence on the bulk refractive-index sensitivity. This behavior allows the resonance characteristics to be electrically adjusted while largely preserving the refractive-index response of the q-BIC modes. To further evaluate the sensing performance by considering both the spectral shift and resonance linewidth, the figure of merit (FOM) is defined as follows [32]:

*FOM* = *S*/*FWHM* (11)

where *FWHM* is the full width at half maximum of the resonance. The *FOM* therefore characterizes the refractive-index-induced frequency shift relative to the resonance linewidth, with a larger value indicating an enhanced capability to resolve small spectral shifts. Figure 7d presents the FOM values of q-BIC I and q-BIC II as functions of δ. Because increasing δ enhances radiative leakage and broadens the q-BIC resonances, the FOM strongly depends on the magnitude of the geometric perturbation. Across the investigated range, the maximum FOM of q-BIC I exceeds 800, whereas that of q-BIC II approaches 10^4^. The substantially higher FOM of q-BIC II originates from the combination of its higher refractive-index sensitivity and narrower resonance linewidth, highlighting its superior potential for high-resolution THz refractive-index sensing.

Table 1 summarizes the refractive-index sensing performance of typical THz quasi-BIC metasurface sensors from recent literature, in comparison with the proposed structure in this work. Among the listed references, the asymmetric metallic aperture q-BIC metasurface [19] is validated by experimental measurements, while the other reference works are based on numerical full-wave electromagnetic simulations. The present work is also a numerical simulation study, consistent with the majority of the compared design-oriented works. Passive quasi-BIC metasurfaces, represented by asymmetric metallic aperture arrays and all-dielectric silicon-strip structures, typically achieve refractive index sensitivities between 147.7 GHz/RIU and 263 GHz/RIU. While these structures benefit from straightforward configuration and no external tuning modules, their operational frequency is permanently fixed after fabrication, which restricts their adaptability to variable sensing scenarios.

Among active tunable schemes, the liquid-crystal-integrated quasi-BIC metasurface enables electrical frequency tuning but yields a relatively low sensitivity of 117 GHz/RIU, and is limited by slow tuning speed and bulky system integration. The graphene-based double-split square ring quasi-BIC sensor delivers a much higher sensitivity of 427 GHz/RIU, demonstrating the outstanding potential of graphene Fermi-level modulation for constructing high-performance tunable THz sensors. Nevertheless, this design only supports single-band operation and lacks polarization-insensitive response, which increases the complexity of optical alignment in practical measurement.

In contrast, the proposed C_4_-symmetric all-dielectric quasi-BIC metasurface with graphene integration simultaneously achieves dual-band operation, electrical tunability, polarization independence and high sensing sensitivity. The q-BIC II mode exhibits a refractive index sensitivity of 475 GHz/RIU, which outperforms all the compared devices listed in the table. In addition, the C_4_ rotational symmetry endows the sensor with identical responses to arbitrarily polarized incident light, and stable sensing performance can be maintained within a wide incident angle range. These merits effectively improve the robustness of the sensing system and avoid the complicated polarization alignment procedure required by conventional polarization-sensitive devices. With the combined advantages of high sensitivity, flexible tunability and excellent operational robustness, the proposed structure provides a promising technical platform for high-performance THz biochemical sensing applications.

## 4. Conclusions

In summary, we numerically demonstrate a graphene-integrated THz q-BIC metasurface based on a C_4_-symmetric supercell that combines high-Q resonances, polarization robustness, electrical tunability, and refractive-index sensing within a single platform. Local geometric perturbations introduced into the individual MMs enable the symmetry-protected states to couple to free space, while the four MMs related by successive 90° rotations preserve the global C_4_ rotational symmetry of the supercell. The resulting q-BIC modes exhibit the characteristic inverse-square dependence of the Q factor on the geometric perturbation. Multipolar analysis further reveals distinct radiation mechanisms for the two modes, with q-BIC I dominated by MQ radiation and q-BIC II primarily governed by TD radiation with an additional MQ contribution. The weaker overall radiative leakage of q-BIC II accounts for its substantially higher Q factor.

The C_4_-symmetric configuration effectively compensates for the orientation-dependent response of the locally anisotropic MMs, resulting in nearly invariant q-BIC spectra for normally incident linearly polarized THz waves over polarization angles from 0° to 90°. Integration of monolayer graphene introduces an additional electrical degree of freedom, enabling continuous modulation of the resonance contrast and Q factor through Fermi-level-dependent conductivity while producing only minor changes in the resonance frequencies and refractive-index sensitivities. For bulk refractive-index sensing, q-BIC I and q-BIC II achieve sensitivities of 345 and 475 GHz/RIU, respectively, with maximum FOM values exceeding 800 and approaching 10^4^. These results establish the C_4_-symmetric q-BIC architecture as a promising strategy for combining polarization-robust resonant sensing with electrically reconfigurable THz functionality.

## Figures and Tables

**Table 1:** Comparison of refractive-index sensing performance of THz q-BIC metasurfaces.

No.	Structure Type	Tuning Mechanism	Refractive Index Sensitivity (GHz/RIU)	Year
1	Liquid-crystal-integrated all-dielectric q-BIC metasurface [22]	Liquid crystal electrical tuning	117	2026
2	Asymmetric metallic aperture q-BIC metasurface [19]	Passive, no tuning	147.7	2023
3	All-dielectric silicon-strip q-BIC metasurface [20]	Passive, no tuning	263	2023
4	Graphene double-split square ring q-BIC metasurface [43]	Graphene Fermi-level tuning	427	2025
5	This work: C_4_-symmetric all-dielectric q-BIC metasurface with graphene	Graphene Fermi-level tuning	345 (q-BIC I)/475 (q-BIC II)	2026

## Data Availability

The original contributions presented in this study are included in the article. Further inquiries can be directed to the corresponding author.
